# Digital evaluation with ABO eCRE of treatment quality and efficiency with completely customized lingual and prefabricated vestibular appliances in combined orthodontic-orthognathic therapy: a retrospective cohort study

**DOI:** 10.1186/s40510-026-00639-w

**Published:** 2026-07-21

**Authors:** Franziska Alina Lang, Norbert Alexander Lang, Gwendolyn Isabella Lode, Frank Hölzle, Rogerio Bastos Craveiro, Michael Wolf

**Affiliations:** 1 Department of Orthodontics, RWTH Aachen University, Aachen, Germany; 2 Department of Oral and Maxillofacial Surgery, RWTH Aachen University, Aachen, Germany

**Keywords:** Combined orthodontic-orthognathic therapy, Maxillo facial surgery, Orthodontic decompensation, Completely customized lingual appliances, Set-Up based appliances, ABO eCRE, Digital quality assessment

## Abstract

**Background:**

Precise orthodontic pre-surgical decompensation is a critical prerequisite for optimal outcomes in orthognathic surgery. However, the relative effectiveness of completely customized lingual appliances (CCLAs) versus prefabricated vestibular multibracket appliances (PVMAs) remains insufficiently established. This study compared the decompensation efficiency, treatment quality, and overall clinical performance of these two appliances.

**Methodology:**

The study included 60 consecutive patients who underwent orthodontic-orthognathic surgery: 30 with CCLA and 30 with PVMA. To ensure quality, the digital ABO CRE for each scanned model was evaluated using OnyxCeph3™ diagnostic software, and the efficiency of each appliance was recorded in the respective patient files. Both groups underwent the same evaluation protocol involving initial, intermediate (pre-surgery) and final casts. An additional set-up model was included for the CCLA group only.

**Results:**

Both groups exhibited comparable baseline scores (mean difference 5.83; 95% CI 1.69 to 9.98). CCLAs demonstrated lower ABO scores than the PVMA group after the decompensation phase (mean difference − 9.23; 95% CI -13.38 to -5.09) and in the final assessment (mean difference − 9.30; 95% CI − 13.44 to -5.16). The largest differences between groups were observed for the alignment criteria in both jaws at the intermediate and final assessments, as well as for all intermaxillary criteria. Treatment efficiency particularly during the decompensation phase, was significantly improved in the CCLA group.

**Conclusion:**

CCLAs demonstrated superior efficiency and higher treatment quality compared with PVMAs in patients undergoing combined orthodontic–orthognathic therapy. The use of CCLAs in combination with orthognathic surgery appears to represent a high-quality treatment approach for correcting pronounced skeletal discrepancies and may serve as a reliable alternative to PVMA in appropriately selected patients.

**Supplementary Information:**

The online version contains supplementary material available at 10.1186/s40510-026-00639-w.

## Introduction

Since the introduction of lingual orthodontic appliances in the 1970s, demand for these appliances has risen significantly in recent years among patients seeking functional and aesthetic improvements [1, 2]. This phenomenon is reflected in the growing number of orthognathic surgeries performed worldwide, as well as a shift towards treating adult patients [3–6]. Interdisciplinary protocols combining orthognathic surgery and orthodontic therapy remain the standard of care for patients with pronounced skeletal dysgnathia and are associated with significant improvements in quality of life [7–9]. Patients typically seek treatment for functional limitations and aesthetic concerns [10, 11]. Occlusal deficiencies are directly correlated with reduced masticatory performance and occlusal stability [12]. The presurgical orthodontic decompensation process has been shown to facilitate enhanced visualisation, alignment of the final occlusion with skeletal correction and occlusal stability after surgery. The subsequent occlusion is characterized by the uniform distribution of forces across all teeth, thereby achieving occlusal harmony. Inadequate decompensation restricts the magnitude of skeletal correction, introduces occlusal interferences that prevent ideal positioning of maxillomandibular segments, and ultimately compromises aesthetics, function, treatment stability and outcome [13]. Because the preoperative occlusion determines—and can limit—surgical bone-segment movements, insufficient preparation risks unstable outcomes and prolonged postoperative treatment [14]. Consequently, efficient and predictable decompensation is pivotal within the orthodontic–orthognathic treatment concept. The concept of completely customized lingual appliances (CCLA) can offer us a potential advantage in the context of preoperative decompensation by providing efficient and predictable treatment [15–20]. This is of central importance within the orthodontic–orthognathic treatment concept. Although the CCLA is well established in the field of orthodontics, there is a lack of documentation or low-level evidence supporting its use in combined orthodontic–surgical protocols [21–25]. However, most studies in the current literature focus on the traditional concept of orthodontic decompensation using prefabricated vestibular multibracket appliances (PVMA), with a particular focus on adjusting the position of the anterior teeth [26–28]. Objective assessments of decompensation quality or therapy outcomes are rarely found in both appliances. Systematic evidence in this specific interdisciplinary context remains limited.

### Specific objectives or hypotheses

In this present study, digital measurements using a modified American Board of Orthodontics electronic Cast Radiographic Evaluation (ABO eCRE) was used for the first time to investigate objectively the quality of treatment of CCLA in comparison with PVMA for combined orthodontic-orthognathic therapy, and to evaluate whether differences in treatment quality and efficiency exist.

## Materials and methods

### Study design

**Fig. 1:**
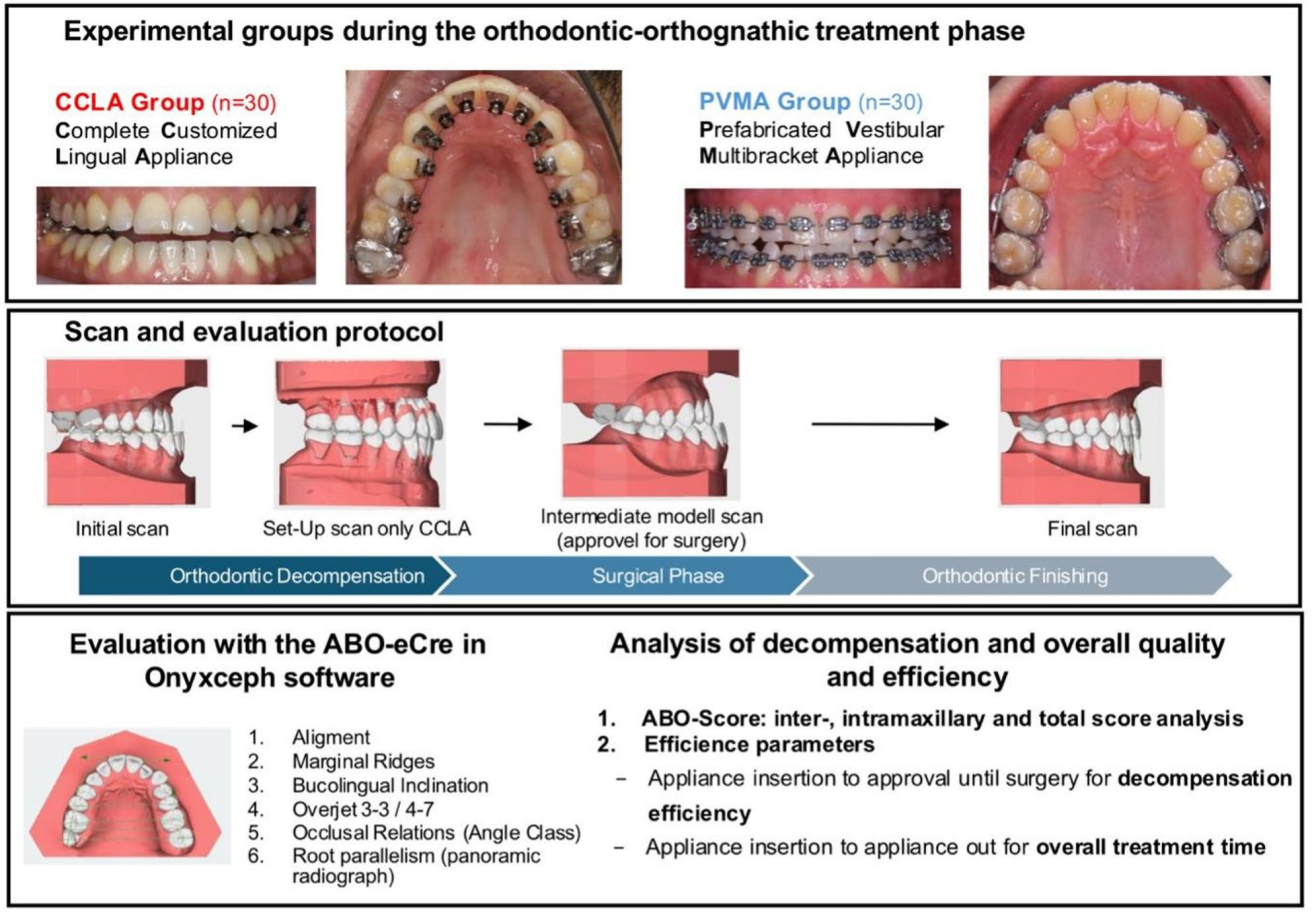
Study design overview. An overview of the study design, including a description of the experimental and control group, the three-dimensional jaw joint measurement and the analysis of lower jaw movements

The present retrospective, pseudonymised, comparative cohort study utilises digital modified ABO eCRE to investigate the potential of CCLA and PVMA approach in combined orthodontic-orthognathic surgery cases to enhance the treatment quality and efficiency. (Fig. 1) This study was conducted in accordance with the principles of the Declaration of Helsinki and was reported according to the STROBE guidelines [29]. (Supplementary Table 1) Ethical approval was obtained from the ethics committee of Medical Faculty of RWTH Aachen with the ethical registration number EK 25–302.

### Participants, eligibility criteria, and setting

This study included patients who underwent consecutive interdisciplinary orthodontic-orthognathic treatment concept at the University Hospital RWTH Aachen between March 2016 and April 2026.

The inclusion criteria were as follows: (1) all patients were aged 18 or older at the time of the surgery; (2) combined therapy involving mono- or bignathic osteotomy and orthodontic treatment; (3) orthodontic decompensation with CCLA or PVMA (4) absence of craniofacial anomalies or abnormal skull shape; (5) absence of facial trauma, mandibular condylar fractures, or maxillary bone defects; (6) complete orthodontic diagnostic data and records; (7) a history of previous orthodontic treatment or missing teeth did not restrict admission. Exclusion criteria: Patients with extensive prosthetic restorations were excluded; Drop out = 0. No patient was excluded due to inadequate oral hygiene or lack of compliance.

### Exposures, predictors, potential confounders, and effect modifiers

After inclusion and exclusion criteria assessment a total of 60 patients were included in the study: Two consecutive treated groups were formed: the CCLA group (*n* = 30; female/male 13/17; mean age 26.00 (7.06) years) and the PVMA group PVMA (*n* = 30; female/male 12/18; mean age 24.34 (7.21) years). An even distribution in terms of age, gender and skeletal class was ensured between both groups.

Potential confounders could affect the therapeutic effect and evaluation of the ABO-Score. These include poor patient compliance, device discomfort and the extent of the surgical procedure. Furthermore, factors such as treatment time were considered effect modifiers, particularly regarding influencing compliance and stress capacity.

### Treatment protocol

In this study, both groups received treatment at our clinic in accordance with standardised protocols for combined orthodontic-orthognathic therapy. This included defined treatment phases, such as preoperative decomposition, surgical intervention and postoperative orthodontic finishing, as well as standard wire sequences adapted to the respective appliance systems.

The CCLA group received treatment using a completely customized, set-up-based lingual bracket system with 0.018-inch slot (WIN, DW Lingual Systems GmbH, Bad Essen, Germany). The standard wire sequence was 0.014-inch nickel-titanium (Ni-Ti), 0.016 × 0.022-inch Ni-Ti, 0.018 × 0.025-inch Ni-Ti, and 0.016 × 0.024 stainless steel (SS) (if required, with 1 cm compression) in the lower jaw, and 0.016 × 0.024 SS with extra torque of 13/21 degrees (canine to canine) and 2 cm expansion in the upper jaw, and 0.018 × 0.018-inch titanium-molybdenum alloy (TMA).

The PVMA cohort was treated with 0.022-inch slot brackets (Legend Standard Brackets, GC Orthodontics, Alsip, Ireland). The wire sequence was as follows: 0.014-inch Ni-Ti; 0.017 × 0.025-inch Ni-Ti; 0.019 × 0.025-inch or 0.021 × 0.025-inch Ni-Ti; 0.019 × 0.025-inch or 0.021 × 0.025 SS; and 0.019 × 0.025-inch TMA. In cases of transverse discrepancy, a transpalatal arch was used in the upper jaw. To achieve the desired individual decompensation for surgical treatment, patients in both cohorts underwent additional decompensation with intermaxillary elastics. No mini-screws or extraoral appliances were used. No extraction was carried out.

Following the surgical procedure, the bimaxillary surgical splint was removed after seven to 14 days. All patients were then treated according to a standardised protocol of intermaxillary elastic fixation, which was gradually reduced from a full mouth ligature until complete removal.

### Digital evaluation workflow

To measure the quality of treatment, the following digitised models were examined in both groups for each patient: Situation before the start of treatment (initial), intermediate model, which represents the surgical model for the manual occlusion planning and after the end of treatment (final). The set-up model was only included in the CCLA group, on which the treatment of patients with completely customized lingual appliances is based (set-up-based treatment). Figure 1 These models were positioned in maximal habitual intercuspation for digitisation. The digitisation was performed using an intraoral scanner (iTero^®^ element™, Align Technology, Inc., Tempe, Arizona, USA or Medit i600, Medit Corp., Seoul, South Korea) [30]. The digital setup model was provided to us by DW Lingual Systems GmbH (Bad Essen, Deutschland). All measurements were carried out by a specialised main examiner and checked by a second expert. The measurements according to the modified digital ABO eCRE (Version Aachen 2026) comprised six components: alignment, marginal ridges, buccolingual inclination, overjet, occlusal relationship and root angulation. The total score includes all six components, including root angulation. The intramaxillary ABO comprises alignment, marginal ridges and buccolingual inclination, and the intermaxillary ABO consists of overjet and occlusal relationship. The set-up models did not include panoramic radiographs to assess root angulation. The digital ABO Score (intramaxillary, intermaxillary and total score) for each scan model was evaluated with a special patch (not freely accessible yet) using the OnyxCeph3TM diagnostic software (Image Instruments GmbH, Chemnitz, Germany), in order to assess the quality of the decompensation and the treatment outcome of both groups.

### Outcomes

The following primary outcome was evaluated: the treatment models of CCLA were compared with those of PVMA in terms of initial complexity, decompensation quality and treatment outcome. The secondary outcome was the comparison of the treatment efficiency. In an additional secondary criterion-based analysis, individual intra- and intermaxillary ABO criteria were analysed to identify which components contributed most to between-group differences in overall treatment quality.

To record the efficiency, the active treatment time, number of appointments (emergency appointments included) and interims diagnostic casts were recorded from both digital and analogue patient files as follows: (1) appliance insertion to approval for surgery (decompensation); (2) appliance insertion to removal excluding the passive time frame approval for surgery to surgical procedure (total active treatment time).

### Reliability

Intra- and interrater reliability was evaluated using intraclass correlation coefficients (ICC). For this purpose, 10 initial models were randomly selected based on the study by Janssens et al., remeasured at least 2 weeks later by the same main examiner (Rater 1), and then independently remeasured by a second control examiner (Rater 2) [31]. ICC estimates were calculated based on a single measurement, absolute-agreement, 2-way mixed effects model. The ICC was calculated using IBM SPSS Statistics 29 (IBM, Armonk, NY) software for the total ABO-Score.

### Sample size calculation

The sample size was not determined using an a priori power analysis, as the CCLA group is a complete survey. All patients treated consecutively with CCLA who met the inclusion criteria were included. The PVMA group was adjusted accordingly based on size.

### Sources of bias and comparability between exposure groups

Although the inclusion criteria were designed to minimise potential bias between the two groups, the different periods over which patients were included could represent a confounding factor. To minimise the risk of selection bias, consecutive cases were included, and no patients were excluded from the analysis for reasons other than those defined in the exclusion criteria. Furthermore, fundamental differences in planning, application and mode of action could represent an additional source of bias.

### Statistical analysis

Statistical analyses were performed using R (version 4.5.2; R Foundation for Statistical Computing, Vienna, Austria) and Prism (version 10.5.0; GraphPad Software). Linear mixed-effects models were fitted with the lme4 package. For the primary outcome, a linear mixed-effects model was used to assess the effects of fixed factors, treatment method (CCLA, PVMA), time (initial, intermediate and final treated as a numeric variable to model linear change over time), score type (intramaxillary, intermaxillary and total ABO scores) and both two-way (including the group × time interaction as the primary effect of interest) as well as the three-way interactions between these factors, including random intercepts by participant. In the criterion-based models, single intra- and intermaxillary ABO criteria (measure) were analysed to identify the components that contributed most to the observed differences between groups. Models were estimated using restricted maximum likelihood (REML) for parameter inference; for comparison of nested random/fixed-effects structures, models were refitted using maximum likelihood (ML) and compared via likelihood ratio tests. Post hoc comparisons of estimated marginal means were conducted using the emmeans package with multiplicity adjustment. Treatment effects are reported as mean differences with 95% confidence intervals, and statistical significance was set at *p* < 0.05. (Supplementary Tables 1,3,5) Model-based contrasts were used to estimate differences between treatment methods at each time point.

Normality was assessed using the Shapiro–Wilk test and visual inspection. Depending on the distributional assumptions, either independent-samples t-tests or Mann–Whitney U tests were applied; both tests were also performed as robustness analyses. All tests were two-sided, and *p* < 0.05 was considered statistically significant. Potential confounding due to baseline differences was assessed by comparing the two appliance groups at the start of treatment (initial).

## Results

### Participant flow and baseline data

**Fig. 2:**
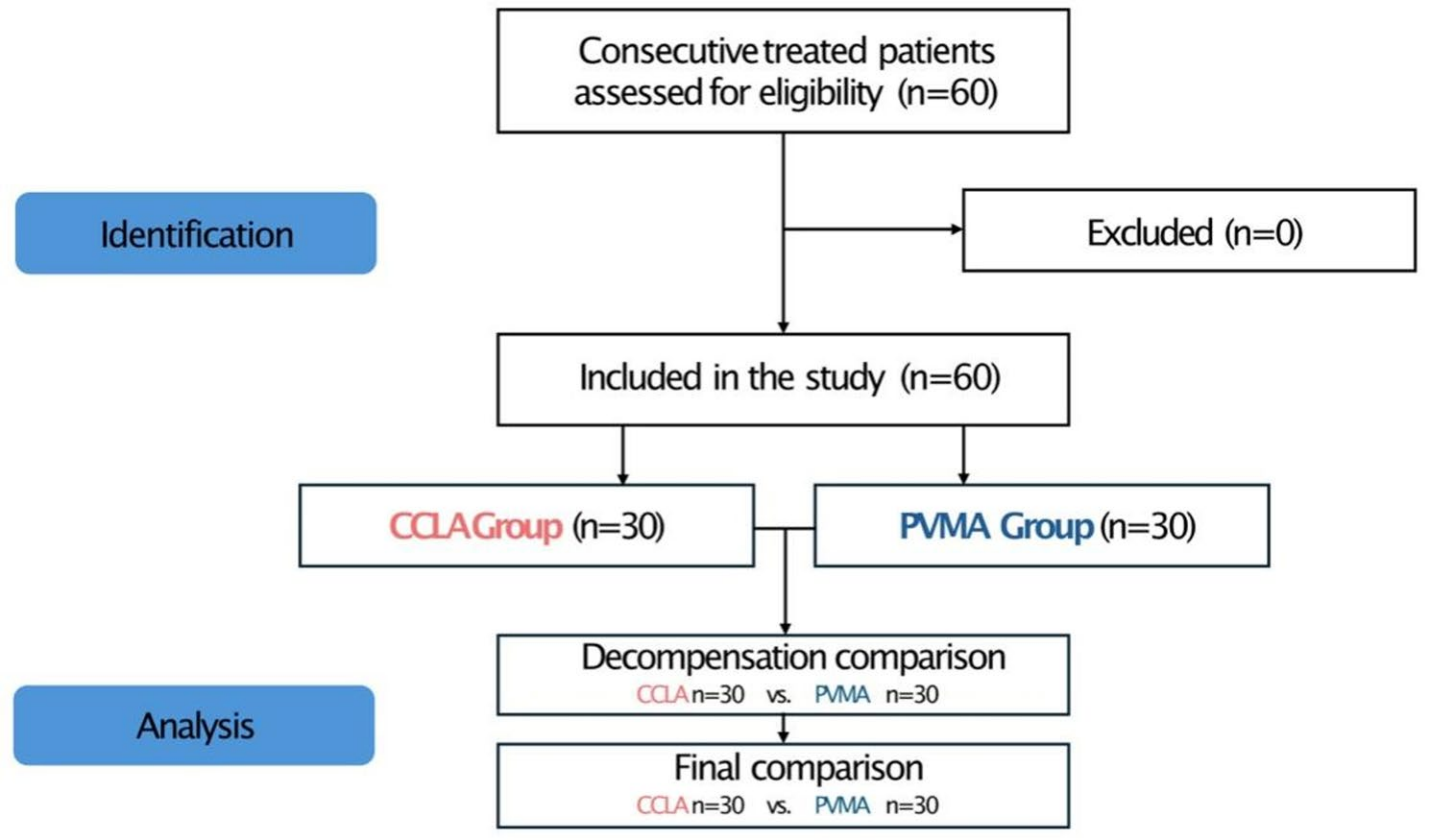
STROBE study flowchart of patient cohort

**Fig. 3:**
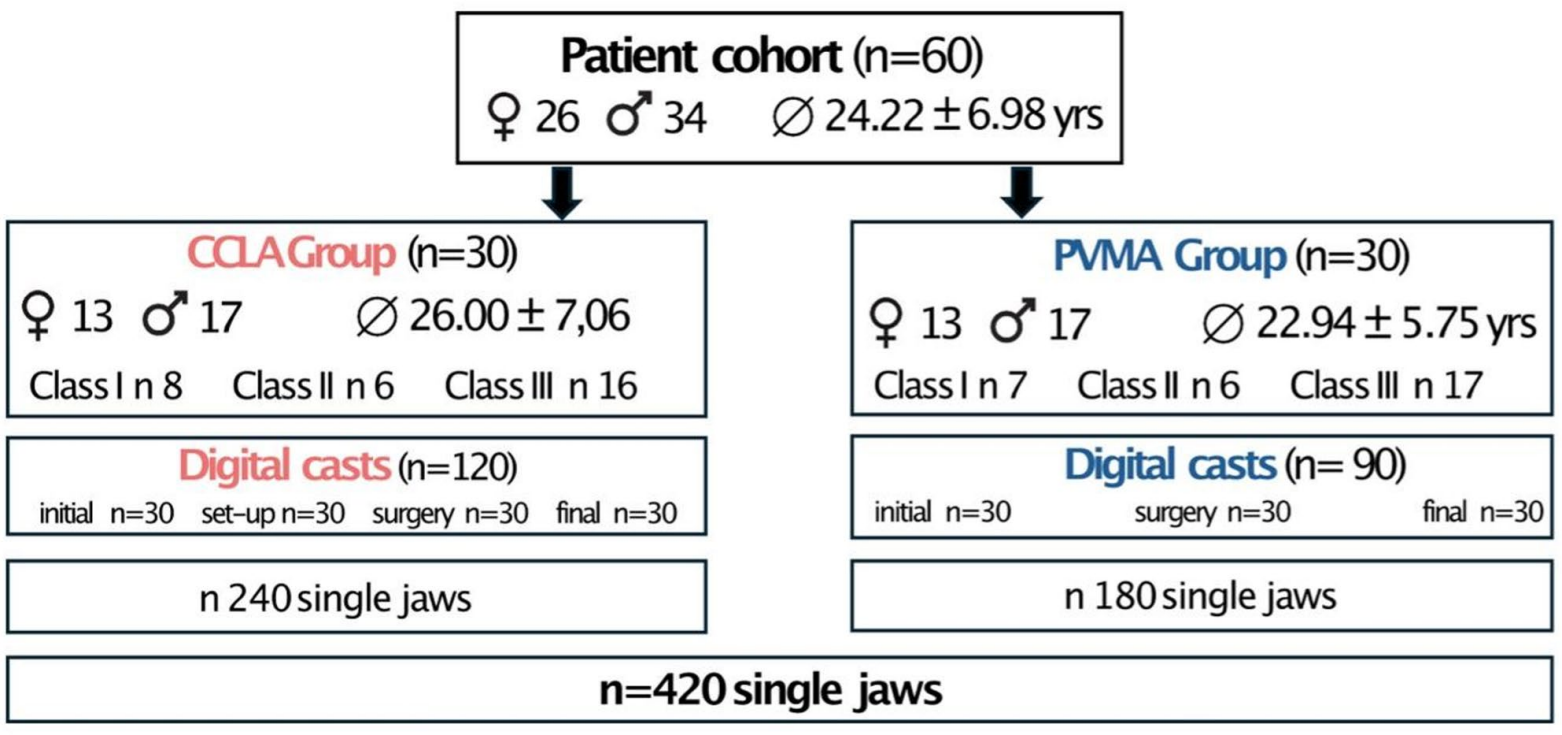
Flowchart of patient cohort and evaluation protocol of the digitised models

Participant flow is reported in the STROBE study flow chart. (Figure 2) A total of 60 adults was treated with CCLA or PVMA and included in the analysis. (Figure 3) A combined orthodontic-orthognathic therapy was undergone by all patients.

### Numbers analysed for each outcome, estimation and precision

Excellent intra- (ICC: 0.907) and inter-rater (ICC: 0.964) reliability was achieved for ten initial models when measuring the total digital ABO score. The correlation coefficients were interpreted according to the specific cut-off limits [32]. Two consecutive treated groups were formed and analysed: the CCLA group (*n* = 30) and its set-up models (*n* = 30) and the PVMA group (*n* = 30) (Fig. 2).

### The CCLA group obtained a better-quality outcome than the PVMA

**Fig. 4:**
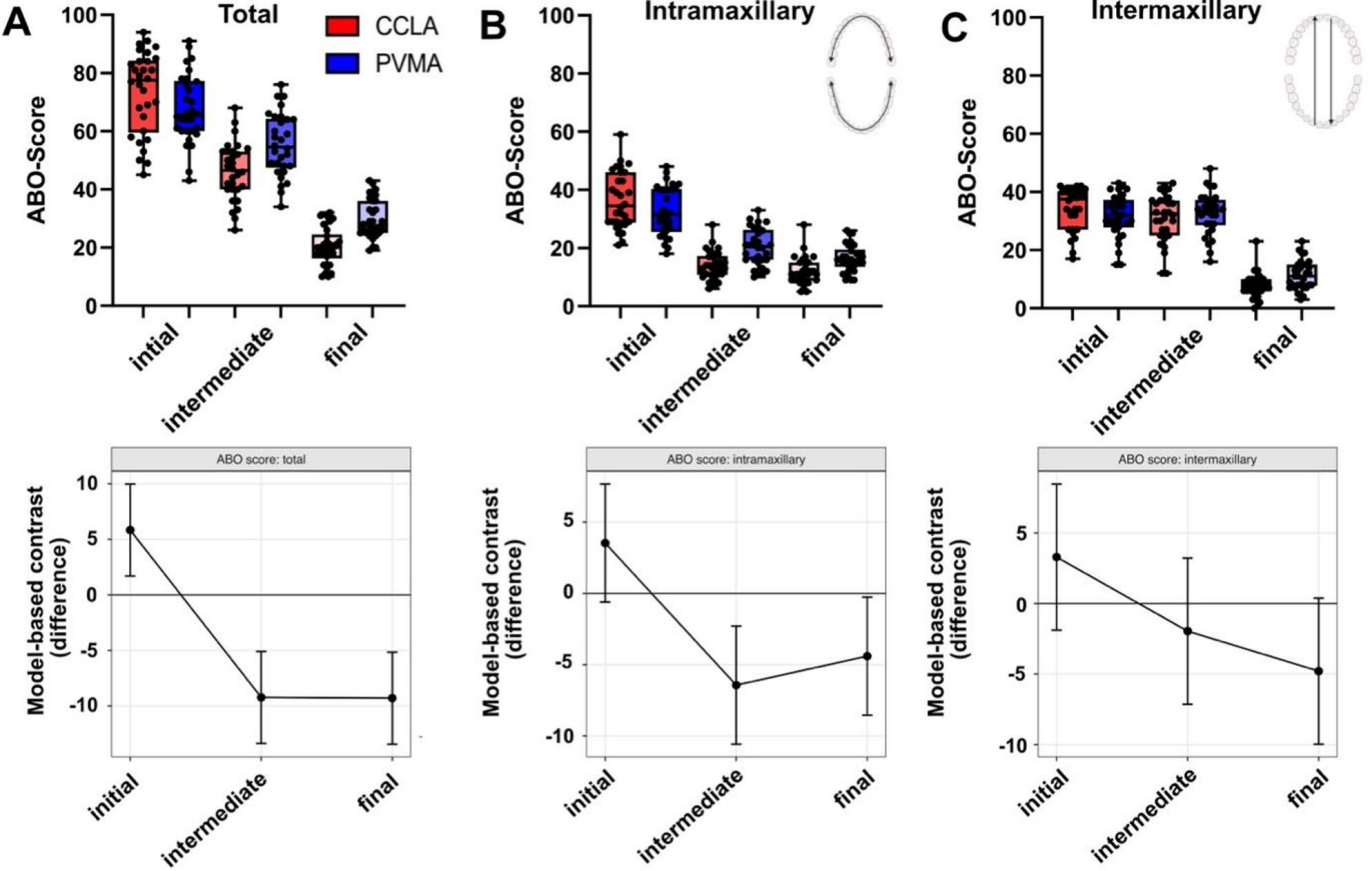
Comparison of the ABO score between CCLA and PVMA at each time point. **A** Comparison of the total,, **B** intramaxillary and, **C** intermaxillary digital ABO scores of CCLA, (red) and PVMA, (blue) for initial, intermediate and final casts, (after debonding). Model-based contrasts between CCLA and PVMA over time for total, intramaxillary, and intermaxillary ABO scores. Points represent estimated mean differences, (CCLA − PVMA) derived from linear mixed-effects models at each time point. Error bars indicate 95% confidence intervals. Negative values indicate lower ABO scores in the CCLA group compared with PVMA. The horizontal reference line at zero denotes no between-group difference

Initially, a comparison of the total ABO scores revealed higher baseline scores for CCLA (73.40 (14.18) vs. PVMA, 67.57 (11.86), mean difference 5.83; 95% CI 1.69 to 9.98) (Fig. 4B). No statistically significant differences between CCLA and PVMA were observed at the initial time point for intramaxillary and intermaxillary ABO scores.

At the intermediate time point, both appliances showed a clear reduction of the intramaxillary ABO score (Fig. 4B). The CCLA group demonstrated significantly lower intramaxillary ABO score compared with the PVMA group (mean difference − 6.43; 95% CI − 10.58 to − 2.29). The total ABO score was also significantly lower in the CCLA group at this timepoint (mean difference − 9.23; 95% CI − 13.38 to − 5.09). No statistically significant difference was found for the intermaxillary score at the intermediate evaluation (Fig. 4C).

At the final assessment, both appliances resulted in an improved final ABO score. The intramaxillary score remained significantly lower in the CCLA group (mean difference − 4.40; 95% CI − 8.54 to − 0.26), and lower in the intermaxillary score (mean difference − 3.83; 95% CI − 7.98 to 0.31). Consequently, the total ABO of CCLA, 20.27 (6.44) and PVMA, 29.57 (6.70) showed a highly significant difference between groups at the end of treatment (mean difference − 9.30; 95% CI − 13.44 to − 5.16). (Fig. 4; Supplementary Fig. 1 and Table 2)

### High congruence between CCLA set-up and final models for intermaxillary ABO score

Most of the reduction in the intramaxillary score for CCLA had been achieved after the decompensation was complete **(**Fig. 4B**)**. In comparison, the intermaxillary score was reduced after surgical correction and showed no noticeable difference to the planned setup in the final model. There was a clear difference in the total ABO score between the final outcome 20.27 (6.44) and the planned setup 13.09 (3.80). The PVMA group’s intermaxillary score remained consistent during the decompensation phase, showing no change from the initial measurement. The PVMA group demonstrated a full reduction of intermaxillary ABO after the decompensation phase (Fig. 4C).

### The CCLA group achieved a significantly better ABO score for alignment and intermaxillary criteria

**Fig. 5:**
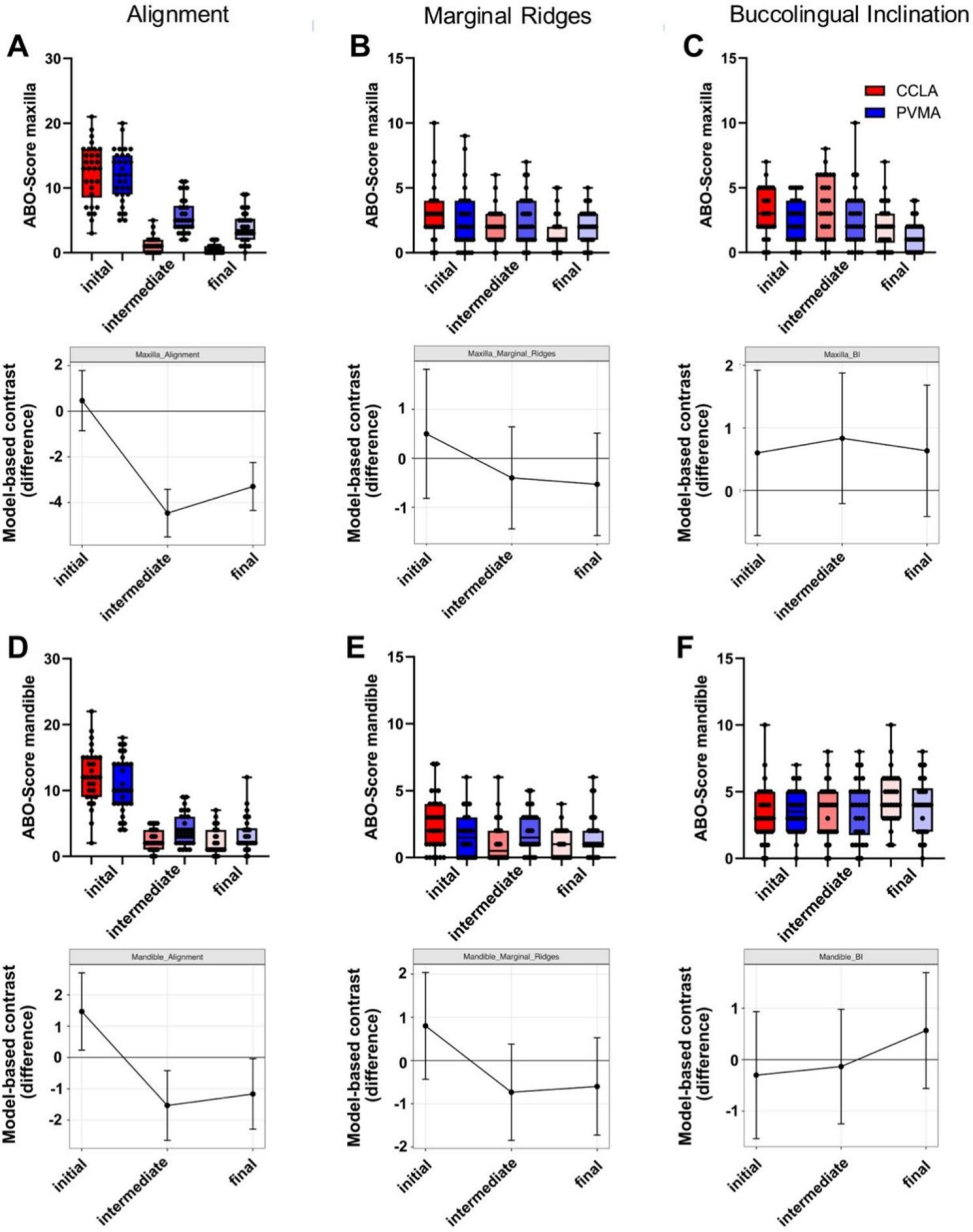
Comparison of the intramaxillary ABO criteria between CCLA and PVMA at each time point. Comparison between CCLA and PVMA in terms of intramaxillary ABO criteria (**A**, **D** alignment; **B**, **C** marginal ridges; **C**, **F** buccolingual inclination). Model-based contrasts between CCLA and PVMA over time for intramaxillary ABO criteria in both jaws. Points represent estimated mean differences (CCLA − PVMA) derived from linear mixed-effects models for alignment, buccolingual inclination (BI), and marginal ridges at each time point. Error bars indicate 95% confidence intervals. Negative values indicate lower ABO scores in the CCLA group compared with PVMA. The horizontal reference line at zero denotes no between-group difference

**Fig. 6:**
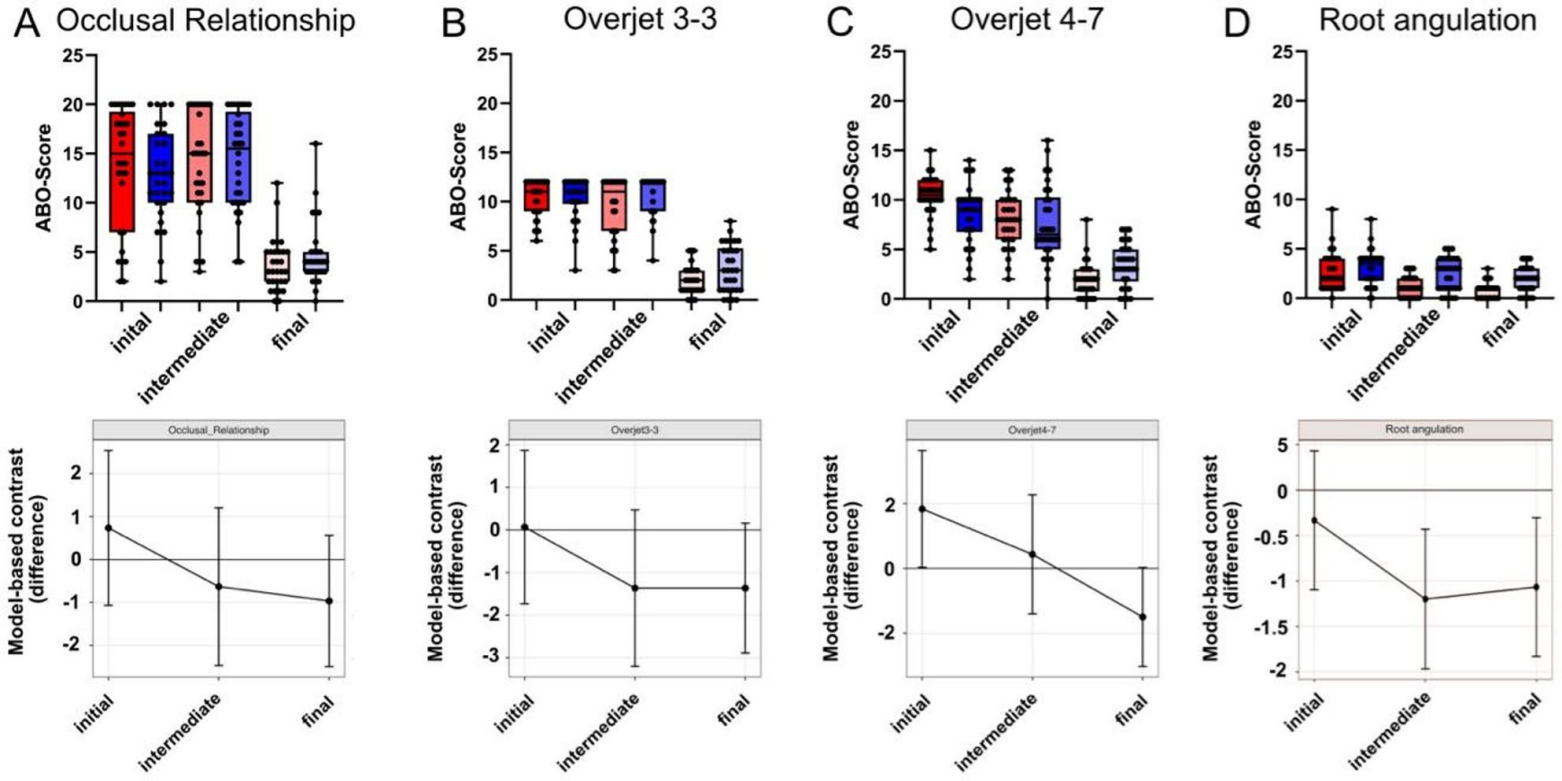
Comparison of the intermaxillary ABO criteria and root angulation between CCLA and PVMA at each time point. Comparison between CCLA and PVMA in terms of intermaxillary ABO criteria (**A** occlusal relationship; **B** overjet 3–3; **C** overjet 4–7) and root angulation (**D**). Model-based contrasts between CCLA and PVMA over time for intermaxillary ABO criteria. Points represent estimated mean differences (CCLA − PVMA) derived from linear mixed-effects models for occlusal relationship, overjet 3–3, overjet 4–7 and root angulation at each time point. Error bars indicate 95% confidence intervals. Negative values indicate lower ABO scores in the CCLA group compared with PVMA. The horizontal reference line at zero denotes no between-group difference

At the initial time point, no significant differences were observed between the CCLA and PVMA groups for any maxillary or mandibular ABO criteria scores, with the exception of alignment in the lower jaw (mean difference 1.47; 95% CI 0.23 to 2.70). At the intermediate evaluation, considerably lower alignment scores were observed in the CCLA group for both the maxilla and the mandible (Fig. 5A, D). In the maxilla, the alignment score was significantly lower in the CCLA group compared with the PVMA group (mean difference − 4.47; 95% CI − 5.51 to − 3.42). Similarly, mandibular alignment scores favoured the CCLA group at the intermediate time point (mean difference − 1.53; 95% CI − 2.65 to − 0.42). No significant differences were found for buccolingual inclination or marginal ridge scores at this stage. The final alignment scores remained significantly lower in the CCLA group for both arches (Fig. 5A, D). In the maxilla, the mean difference in alignment score was − 3.30 (95% CI − 4.35 to − 2.25), while in the mandible a mean difference of − 1.17 points (95% CI − 2.29 to − 0.04) were observed. No significant differences between groups were detected for buccolingual inclination or marginal ridges in either the maxilla or mandible at the final time point. (Fig. 5; Supplementary Fig. 2 and Table 4) No significant differences were observed between the CCLA and PVMA groups for any of the intermaxillary criteria. (Fig. 6; Supplementary Fig. 3 and Table 6) Analysis of the panoramic radiograph revealed that the root angulation value was significantly lower in the CCLA following the decompensation phase (mean difference − 1.20; 95% CI -1.97 to -0.43) and at the end of treatment (mean difference − 1.07; 95% CI -1.83 to -0.30). (Fig. 6; Supplementary Table 6)

### The CCLA group completed the decompensation phase and overall active treatment faster

**Fig. 7:**
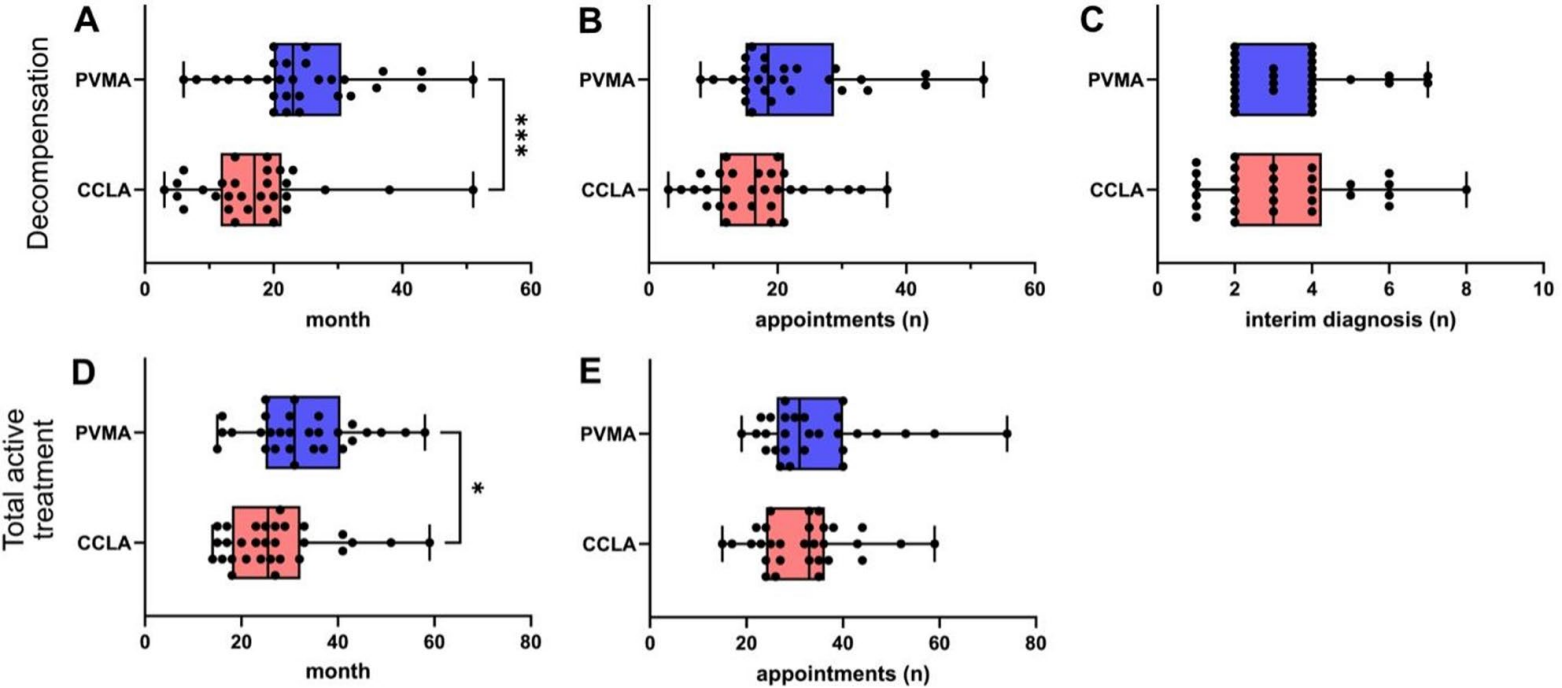
Comparison of the treatment efficiency between CCLA and the PVMA. Representation of treatment efficiency during preoperative orthodontic decompensation (appliance insertion to approval for surgery) of both treatment groups: **A** time of decompensation in months; **B** number of appointments; **C** number of interim diagnoses; and total active treatment (approval for surgery to surgical procedure excluded): **D** total time in months; **E** number of total appointments

A comparison of treatment duration during the decompensation phase showed that CCLA treatment was significantly shorter than PVMA treatment (17.23 (9.81) months vs. 24.83 (10.31) months; Fig. 7A). Consistent with this finding, fewer appointments were required for CCLA treatment than for PVMA treatment (16.87 (8.18) vs. 22.36 (10.58), Fig. 7B). The number of orthodontic interim diagnoses was comparable in both groups (Fig. 7C). Overall, the CCLA group required a significantly shorter treatment time (27.20 (10.87) months vs. PVMA, 32.72 (11.00) months) and fewer appointments (31.97 (9.92) vs. PVMA, 34.54 (11.65)) during the active treatment phase (Fig. 7D, E).

## Discussion

### Main findings in the context of the existing evidence, interpretation

The demand for new orthodontic appliances has increased significantly among patients who wish to improve their functional occlusion and receive higher medical quality. To achieve this, it is necessary to evaluate the quality and efficiency of treatment, with the aim of improving it and developing more modern methods [33]. Recent studies, which have so far focused on comparing conservative therapies, have not established any differences in treatment quality between customized lingual appliances (CCLA) and prefabricated vestibular multibracket appliances (PVMA) [34–37]. PVMA has proved to be an effective device for treating orthodontic patients. It is considered the gold standard for combined orthodontic- orthognathic treatment [38–40]. Although CCLAs are widely used in conservative orthodontic treatment [19, 34, 41], they have only been described in a limited number in treatment protocols combining orthodontic and surgical components [21–25, 42]. In the context of orthodontic-orthognathic treatment, no comparison of the quality of treatment between CCLA and PVMA could be found. The initial difficulties encountered during the clinical introduction of CCLA, may prevent their widespread use. These difficulties include the practical challenges of handling the appliances, longer treatment times for patients and orthodontists, higher laboratory costs, poorer patient comfort and poorer results compared to labial appliances [2, 43, 44]. However, advances in development and laboratory techniques, coupled with the widespread use of sophisticated computer programs, have reintroduced lingual appliances as a promising and competitive technology [2, 43, 45]. The number of publications on this topic has increased steadily over the last decade, reflecting the growing interest among dental professionals. At the same time, demand for these appliances has risen significantly among patients seeking aesthetic improvements [2].

Combined orthodontic–orthognathic treatment was selected as the clinical setting for this study because it follows a highly standardised treatment protocol, including defined treatment stages, appliance-specific archwire sequences, pre-surgical orthodontic decompensation, surgical correction and post-surgical finishing. In addition, the final intermaxillary relationship is largely determined by the surgical skeletal correction itself, which reduces the influence of patient compliance and other confounding variables compared with non-surgical orthodontic treatment. This provides a comparatively homogeneous and controlled cohort for evaluating quality between different treatment concepts. In this context, precise pre-surgical decompensation and intramaxillary alignment are essential to enable accurate surgical repositioning and stable postoperative occlusion.

To objectively evaluate and compare two treatment concepts regarding decompensation and treatment quality, strict and precise quantification of individual tooth positions, among many other factors, must be carried out. In our study, we opted for the ABO CRE because, although it is more complex, it provides a more robust evaluation than the Peer Assessment Rating (PAR) [46]. The PAR is an effective, relatively simple assessment that primarily focuses on the treatment effect and the overall situation rather than the positioning of individual teeth and overemphasises overjet [47]. The ABO-OGS/CRE framework is therefore more suitable for this study’s specific aims, as it enables detailed qualitative and quantitative assessments of deviations from ideal occlusion across dental arches, without applying weighting factors to selected components. Given the surgical indication, all patients in this cohort presented with clinically relevant skeletal discrepancies, which were primarily addressed by orthognathic surgery. The baseline evaluation therefore focused on dental malocclusion rather than on skeletal discrepancy severity. Baseline ABO-eCRE scores were used to quantify the deviation from ideal occlusion, with a score of 0 representing the ideal treatment goal, and to assess comparability between the two treatment concepts. The high baseline scores in both groups reflected substantial dental deviations before treatment. The comparable values between groups indicate that the extent of these deviations was similar at baseline. This methodological approach was adopted for research in this field [19, 31, 48, 49].

The high congruence between CCLA set-up and final models has already been confirmed in the literature [15, 19, 31, 50]. In addition, the intermaxillary parameters showed a high level of comparability with the planned target set-up. Two things are required for this tooth movement: sufficient force and torque control to minimise tilting. This can be achieved using a completely customized appliance that implements the planned tooth positions of the planned alignment precisely [15, 16, 20]. Conventional standardised brackets are manufactured according to predefined prescriptions and dimensions. However, variations in slot dimensions, bracket fit and surface characteristics are unavoidable, which can restrict precise tooth movement and low torque transmission. Consequently, additional wire bending and finishing adjustments are often necessary to compensate for these inaccuracies and achieve the desired outcome [51, 52]. CCLAs are manufactured in accordance with an individual target set-up and specific objectives, allowing the planned tooth positions to be directly incorporated into the bracket design. The high clinical reliability reported for this approach is largely attributed to the exceptional manufacturing precision of CCLAs, in particular the high-precision milled bracket slots with minimal tolerances of only 0.1% [20], and through the custom-made archwires. The question of whether further improvement of the score close to the setup could be clinically relevant remains open.

Adequate dental decompensation is essential for successful treatment in a combined procedure [27]. The CCLA’s superior alignment capability and precise torque transmission [16, 18, 20, 53] during the pre-surgical decompensation phase eliminate potential occlusal interferences that could prevent the planned final occlusion. This would enable complete interdigitation at the desired postoperative overjet and overbite, ensuring the stability of the skeletal segments [14, 27]. Inadequate decompensation restricts the magnitude of skeletal correction and introduces occlusal interferences that prevent the maxillomandibular segments from being positioned ideally. This ultimately compromises aesthetics, function, treatment stability and outcome [13, 27]. The overjet of the front teeth remained similar with both appliances. It is common in orthodontic decompensation for the overjet to either remain unchanged or become more pronounced, to allow optimal movement of the osteotomy fragments. The higher decompensation quality and more precise individual tooth movement, as determined by the setup, suggest a lower final ABO score and superior treatment outcome for CCLA.

Deguchi and colleagues observed poorer root parallelism with lingual appliances, particularly in the anterior region [37]. The reduced lingual interbracket distance compared to vestibular appliances was found to result in poor root alignment. However, more recent findings show similar OGS scores between set-up-based lingual appliances and prefabricated vestibular multibracket appliances [35, 36]. It should be noted here that most studies involved treatment with different vestibular and lingual systems. The long-established and carefully refined indirect bonding protocol may have facilitated the precise intraoral transfer of planned bracket positions [54], thereby supporting improved root control and root paralleling on panoramic radiographs. Particularly with direct bracket bonding, inaccuracies may occur depending on the practitioner’s experience.

The clinical relevance of the effects is particularly noteworthy at the final stage: an average difference of 10 ABO points in favour of the lingual appliance clearly falls within the range of a treatment-relevant outcome [31, 48]. This is largely determined by the improved results in the intramaxillary criterion of alignment in both jaws, as well as the improved results in the intermaxillary criteria of frontal and lateral overjet following surgical correction. In our view, this can be attributed to the concept of the completely customized lingual appliance, which involves setup-based planning utilising highly precise bracket slots and perfectly fitting customized archwires.

Treatment with CCLA resulted in a shorter decompensation phase than with PVMA, requiring fewer appointments and consequently reducing the overall treatment time. However, the post-operative phase of orthodontic finishing was shorter for PVMA. This may contradict the assumption that more precise and faster preparation in relation to the decompensation phase can also accelerate the final phase. However, the psychological stress caused by procedures such as orthognathic surgery should not be ignored, nor should the fatigue that sets in after long periods of treatment [55, 56]. This can have a significant impact on patient compliance and may lead to premature termination of therapy, which may no longer be determined by the professional competence of the orthodontic specialist. It should be noted that compliance of the patient cohort was not an exclusion criterion in our study. A delay in achieving the desired result may also be due to patients forgetting to wear elastic, which are important for postoperative intermaxillary fixation and settling the teeth after surgery. Forgetting is not uncommon, and alongside pain, laziness and embarrassment, it is one of the most common reasons for not wearing elastics [57].

### Limitations

The present study focuses primarily on the quality of treatment, disregarding its functional and aesthetic impact on patients. It seems important to include patient perceptions in future studies. Furthermore, the type of surgical intervention was not considered, which could potentially affect both compliance and treatment quality. Completely customized lingual appliances naturally require a workflow based on a set-up. Although the use of a set-up-based approach for vestibular appliances could influence treatment outcomes, this does not correspond to our current clinical practice. Furthermore, there are currently no data available on completely customized vestibular appliances in the context of orthognathic treatment.

### Generalisability

It should be noted that the ABO score obtained using this software-based ABO-eCRE may differ from that obtained using the analogue version. This could limit the generalisability of the findings for users of the analogue version or other digital programmes. In this study, the digitally assessed score is used to compare the quality of different treatment approaches. The patients in this study were treated at one orthodontic centre in Germany. Therefore, the results may not be fully generalisable to other orthodontic settings.

## Conclusions

In conclusion, completely customized lingual appliances demonstrate superior clinical outcomes and significantly higher treatment efficiency compared with prefabricated vestibular multibracket appliances in combined orthodontic–orthognathic therapy. The treatment concept using completely customized lingual appliances in combination with orthognathic surgery appears to be an effective and high-quality approach for correcting pronounced skeletal discrepancies. This may serve as a reliable alternative to prefabricated vestibular multibracket appliances in appropriately selected patients.

## Supplementary Information

Below is the link to the electronic supplementary material.

Supplementary Material 1.

Supplementary Material 2.

## Data Availability

The data supporting the findings of this study are available from the corresponding author upon request.
