# Supplementary material for: Digital evaluation with ABO eCRE of treatment quality and efficiency with completely customized lingual and prefabricated vestibular appliances in combined orthodontic-orthognathic therapy: a retrospective cohort study
Source: Prog Orthod. 2026 Jul 21;27:36. doi: 10.1186/s40510-026-00639-w (PMC13388873; doi:10.1186/s40510-026-00639-w)
Supplement: Supplementary file 1 — Supplementary Material 1. [file 40510_2026_639_MOESM1_ESM.docx]

**Supplementary**

**Supplementary Table 1**. Estimates of ABO score over time using a linear mixed-effects model

|  | | | |
| --- | --- | --- | --- |
| **Characteristic** | **Beta** | **95% CI** | **p-value** |
| method |  |  |  |
| PVMA | — | — |  |
| CCLA | 1.5 | -2.7, 5.8 | 0.5 |
| time_num | -8.3 | -10, -6.2 | <0.001 |
| score_type |  |  |  |
| intramaxillary | — | — |  |
| intermaxillary | 3.9 | 0.21, 7.6 | 0.038 |
| total | 38 | 35, 42 | <0.001 |
| method * time_num |  |  |  |
| CCLA * time_num | -4.0 | -6.8, -1.1 | 0.007 |
| method * score_type |  |  |  |
| CCLA * intermaxillary | 0.78 | -4.5, 6.0 | 0.8 |
| CCLA * total | 1.8 | -3.4, 7.0 | 0.5 |
| time_num * score_type |  |  |  |
| time_num * intermaxillary | -1.9 | -4.8, 0.97 | 0.2 |
| time_num * total | -11 | -14, -7.9 | <0.001 |
| method * time_num * score_type |  |  |  |
| CCLA * time_num * intermaxillary | 0.73 | -3.3, 4.8 | 0.7 |
| CCLA * time_num * total | -3.6 | -7.7, 0.46 | 0.082 |

Abbreviation: CI = Confidence Interval

Fixed effects included treatment method (CCLA vs PVMA), time (treated as a numeric variable), score type (intramaxillary, intermaxillary, and total), and all two-way and three-way interactions. Estimates are reported as beta coefficients with 95% confidence intervals and p-values. PVMA and intramaxillary score served as reference categories. Negative beta values indicate lower ABO scores over time or in the CCLA group relative to PVMA.

**Supplementary Table 2.** Model-based contrasts of the ABO score over time

| **Model-based contrasts CCLA - PVMA** | | | | |
| --- | --- | --- | --- | --- |
| **score-type** | **time** | **effect-size** | **95% CI of diff.** | **P-value** |
| total | initial | 5.833 | 5.83 (1.69, 9.98) | 0.006 |
|  | intermediate | -9.233 | -9.23 (-13.38, -5.09) | 0.000 |
|  | final | -9.300 | -9.30 (-13.44, -5.16) | 0.000 |
| intramaxillary | initial | 3.533 | 3.53 (-0.61, 7.68) | 0.094 |
|  | intermediate | -6.433 | -6.43 (-10.58, -2.29) | 0.002 |
|  | final | -4.400 | -4.40 (-8.54, -0.26) | 0.037 |
| intermaxillary | initial | 2.633 | 2.63 (-1.51, 6.78) | 0.212 |
|  | intermediate | -1.567 | -1.57 (-5.71, 2.58) | 0.457 |
|  | final | -3.833 | -3.83 (-7.98, 0.31) | 0.070 |

Differences between CCLA and PVMA are shown for intramaxillary, intermaxillary, and total ABO scores at initial, intermediate, and final time points. Effect sizes are presented as mean differences with corresponding 95% confidence intervals (CI) and results of the linear mixed model analyses (*p* value).

**Supplementary Table 3.** Estimates of intramaxillary ABO criteria over time using a linear mixed-effects model

| **Characteristic** | **Beta** | **95% CI** | **p-value** |
| --- | --- | --- | --- |
| group |  |  |  |
| PVMA | — | — |  |
| CCLA | -0.55 | -1.7,0.62 | 0.4 |
| time_num | -4.0 | -4.6, -3.3 | <0.001 |
| measure |  |  |  |
| Maxilla_Alignment | — | — |  |
| Maxilla_BI | -8.5 | -9.6, -7.4 | <0.001 |
| Maxilla_Marginal_Ridges | -8.6 | -9.8, -7.5 | <0.001 |
| group * time_num |  |  |  |
| CCLA * time_num | -1.9 | -2.8, -1.0 | <0.001 |
| group * measure |  |  |  |
| CCLA * Maxilla_BI | 1.2 | -0.39, 2.8 | 0.14 |
| CCLA * Maxilla_Marginal_Ridges | 0.92 | -0.69, 2.5 | 0.3 |
| time_num * measure |  |  |  |
| time_num * Maxilla_BI | 3.5 | 2.6, 4.3 | <0.001 |
| time_num * Maxilla_Marginal_Ridges | 3.8 | 2.9, 4.7 | <0.001 |
| group * time_num * measure |  |  |  |
| CCLA * time_num * Maxilla_BI | 1.9 | 0.65, 3.2 | 0.003 |
| CCLA * time_num * Maxilla_Marginal_Ridges | 1.4 | 0.12, 2.6 | 0.032 |

| **Characteristic** | **Beta** | **95% CI** | | **p-value** |
| --- | --- | --- | --- | --- |
| group |  | |  |  |
| PVMA | — | | — |  |
| CCLA | 0.91 | | -0.27, 2.1 | 0.13 |
| time_num | -3.7 | | -4.3, -3.1 | <0.001 |
| measure |  | |  |  |
| Mandible_Alignment | — | | — |  |
| Mandible_BI | -6.2 | | -7.3, -5.0 | <0.001 |
| Mandible_Marginal_Ridges | -8.0 | | -9.1, -6.9 | <0.001 |
| group * time_num |  | |  |  |
| CCLA * time_num | -1.3 | | -2.2, -0.44 | 0.003 |
| group * measure |  | |  |  |
| CCLA * Mandible_BI | -1.3 | | -2.9, 0.31 | 0.11 |
| CCLA * Mandible_Marginal_Ridges | -0.38 | | -2.0, 1.2 | 0.6 |
| time_num * measure |  | |  |  |
| time_num * Mandible_BI | 3.9 | | 3.0, 4.8 | <0.001 |
| time_num * Mandible_Marginal_Ridges | 3.7 | | 2.8, 4.6 | <0.001 |
| group * time_num * measure |  | |  |  |
| CCLA * time_num * Mandible_BI | 1.8 | | 0.50, 3.0 | 0.006 |
| CCLA * time_num * Mandible_Marginal_Ridges | 0.62 | | -0.63, 1.9 | 0.3 |
| Abbreviation: CI = Confidence Interval | | | | |

Separate models were fitted for maxillary and mandibular criteria. Fixed effects included treatment group (CCLA vs PVMA), time (treated as a numeric variable), measure (alignment, buccolingual inclination, and marginal ridges), and all two-way and three-way interactions. Random intercepts (and random slopes for time) were included at the participant level. Estimates are reported as beta coefficients with 95% confidence intervals (CI) and p-values. PVMA and alignment served as reference categories. Negative beta values indicate lower ABO scores over time or in the CCLA group relative to PVMA.

**Supplementary Table 4.** Model-based contrasts of the intramaxillary ABO criteria over time

| **Model-based contrasts CCLA - PVMA** | | | | | |
| --- | --- | --- | --- | --- | --- |
| **Maxilla** | | | | | |
| **criteria** | **time** | **effect-size** | | **95% CI of diff.** | **P-value** |
| Alignment | initial | | 0.467 | 0.47 (-0.85, 1.79) | 0.486 |
|  | intermediate | | -4.467 | -4.47 (-5.51, -3.42) | 0.000 |
|  | final | | -3.300 | -3.30 (-4.35, -2.25) | 0.000 |
| Buccolingual inclination | initial | | 0.600 | 0.60 (-0.72, 1.92) | 0.371 |
|  | intermediate | | 0.833 | 0.83 (-0.21, 1.88) | 0.117 |
|  | final | | 0.633 | 0.63 (-0.42, 1.68) | 0.236 |
| Marginal Ridges | initial | | 0.500 | 0.50 (-0.82, 1.82) | 0.456 |
|  | intermediate | | -0.400 | -0.40 (-1.44, 0.64) | 0.451 |
|  | final | | -0.533 | -0.53 (-1.58, 0.52) | 0.318 |
| **Mandible** | | | | | |
| **criteria** | **time** | | **effect-size** | **95% CI of diff.** | **P-value** |
| Alignment | initial | | 1.467 | 1.47 (0.23, 2.70) | 0.020 |
|  | intermediate | | -1.533 | -1.53 (-2.65, -0.42) | 0.007 |
|  | final | | -1.167 | -1.17 (-2.29, -0.04) | 0.042 |
| Buccolingual inclination | initial | | -0.300 | -0.30 (-1.53, 0.93) | 0.632 |
|  | intermediate | | -0.133 | -0.13 (-1.25, 0.98) | 0.814 |
|  | final | | 0.567 | 0.57 (-0.56, 1.69) | 0.323 |
| Marginal Ridges | initial | | 0.800 | 0.80 (-0.43, 2.03) | 0.203 |
|  | intermediate | | -0.733 | -0.73 (-1.85, 0.38) | 0.196 |
|  | final | | -0.600 | -0.60 (-1.73, 0.53) | 0.295 |

Differences between CCLA and PVMA are shown for intramaxillary criteria Alignment, Buccolingual Inclination and Marginal Ridges at initial, intermediate, and final time points. Effect sizes are presented as mean differences with corresponding 95% confidence intervals (CI) and results of the linear mixed model analyses (*p* value).

**Supplementary Table 5.** Estimates of intermaxillary ABO criteria over time using a linear mixed-effects model

| **Characteristic** | **Beta** | **95% CI** | **p-value** |
| --- | --- | --- | --- |
| group |  |  |  |
| PVMA | — | — |  |
| CCLA | 0.56 | -1.2, 2.3 | 0.5 |
| time_num | -4.1 | -5.1, -3.2 | <0.001 |
| measure |  |  |  |
| Occlusal_Relationship | — | — |  |
| Overjet3-3 | -3.1 | -4.8, -1.4 | <0.001 |
| Overjet4-7 | -5.7 | -7.4, -4.0 | <0.001 |
| group * time_num |  |  |  |
| CCLA * time_num | -0.85 | -2.2, 0.47 | 0.2 |
| group * measure |  |  |  |
| CCLA * Overjet3-3 | -0.73 | -3.1, 1.7 | 0.6 |
| CCLA * Overjet4-7 | 1.4 | -1.1, 3.8 | 0.3 |
| time_num * measure |  |  |  |
| time_num * Overjet3-3 | 0.65 | -0.67, 2.0 | 0.3 |
| time_num * Overjet4-7 | 1.6 | 0.23, 2.9 | 0.022 |
| group * time_num * measure |  |  |  |
| CCLA * time_num * Overjet3-3 | 0.13 | -1.7, 2.0 | 0.9 |
| CCLA * time_num * Overjet4-7 | -0.82 | -2.7, 1.1 | 0.4 |

Fixed effects included treatment method (CCLA vs PVMA), time (treated as a numeric variable), measure (Occlusal Relationship, Overjet 3-3, Overjet 4-7), and all two-way and three-way interactions. Estimates are reported as beta coefficients with 95% confidence intervals and p-values. PVMA and intramaxillary score served as reference categories. Negative beta values indicate lower ABO scores over time or in the CCLA group relative to PVMA.

**Supplementary Table 6.** Model-based contrasts of the intermaxillary and root angulation ABO criteria over time

| **Model-based contrasts CCLA - PVMA** | | | | |
| --- | --- | --- | --- | --- |
| **Maxilla & Mandible** | | | | |
| **criteria** | **time** | **effect-size** | **95% CI of diff.** | **P-value** |
| Occlusal Relationship | initial | 0.733 | 0.73 (-1.07, 2.53) | 0.423 |
|  | intermediate | -0.633 | -0.63 (-2.47, 1.20) | 0.497 |
|  | final | -0.967 | -0.97 (-2.49, 0.56) | 0.214 |
| Overjet 3-3 | initial | 0.067 | 0.07 (-1.73, 1.87) | 0.942 |
|  | intermediate | -1.367 | -1.37 (-3.20, 0.47) | 0.144 |
|  | final | -1.367 | -1.37 (-2.89, 0.16) | 0.079 |
| Overjet 4-7 | initial | 1.833 | 1.83 (0.03, 3.63) | 0.046 |
|  | intermediate | 0.433 | 0.43 (-1.40, 2.27) | 0.642 |
|  | final | -1.500 | -1.50 (-3.03, 0.03) | 0.054 |
|  | initial | -0.333 | -0.33 (-1.10, 0.43) | 0.389 |
| Root angulation | intermediate | -1.199 | -1.20 (-1.97, -0.43) | 0.002 |
|  | final | -1.067 | -1.07 (-1.83, -0.30) | 0.006 |

Differences between CCLA and PVMA are shown for the intermaxillary criteria Occlusal Relationship, Overjet 3-3, Overjet 4-7 and Root angulation at initial, intermediate, and final time points. Effect sizes are presented as mean differences with corresponding 95% confidence intervals (CI) and results of the linear mixed model analyses (*p* value).

**Supplementary Figure 1.** Model-estimated marginal means of ABO score over time by treatment group


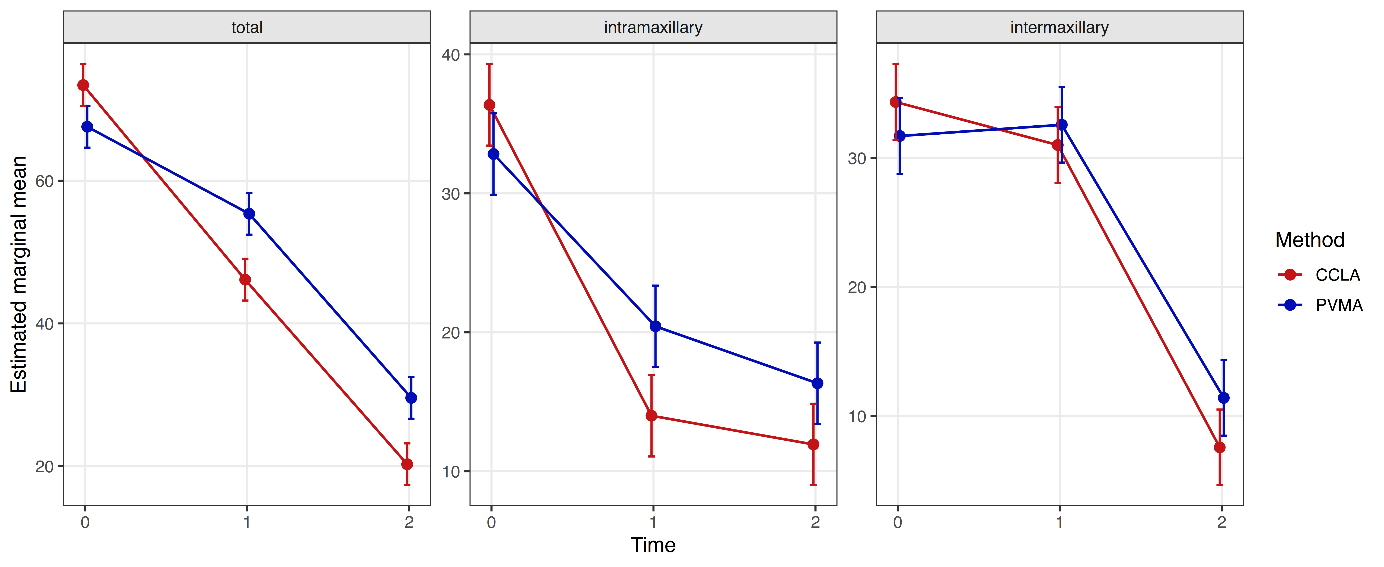


Estimated marginal means with 95% confidence intervals for total, intramaxillary and inter-maxillary ABO scores at initial (0), intermediate (1) and final (2) assessment time points for both treatment groups. The model-based mean estimates are represented by the points, and the error bars show the 95% confidence intervals. Lower ABO scores indicate better treatment quality.

**Supplementary Figure 2.** Model-estimated marginal means of intramaxillary ABO criteria over time by treatment group


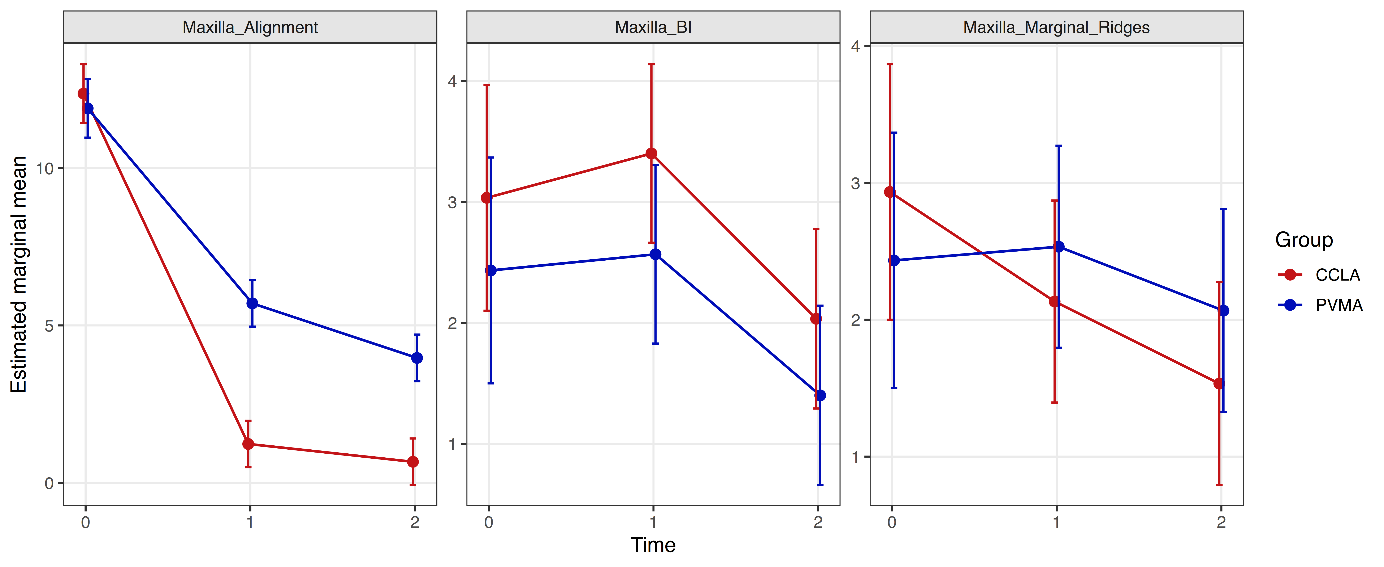

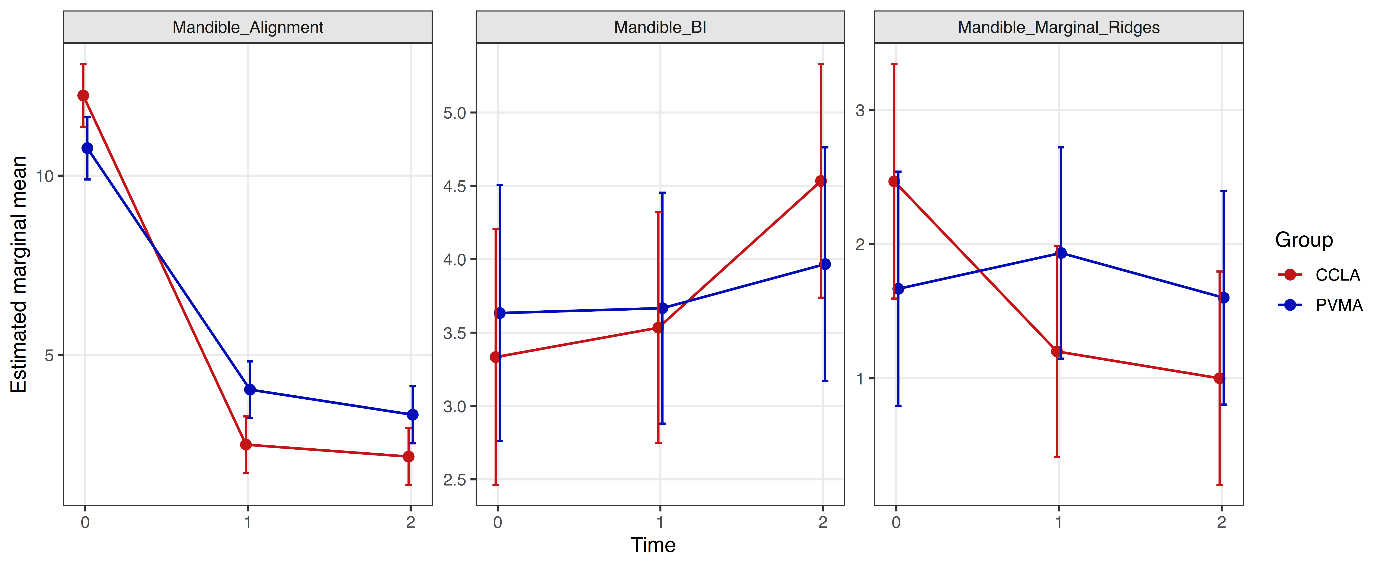


Estimated marginal means with 95% confidence intervals for the intramaxillary ABO criteria of the maxilla and mandible at initial (0), intermediate (1) and final (2) assessment time points for both treatment groups. The model-based mean estimates are represented by the points, and the error bars show the 95% confidence intervals. Lower ABO scores indicate better treatment quality.

**Supplementary Figure 3.** Model-estimated marginal means of intermaxillary ABO criteria over time by treatment group


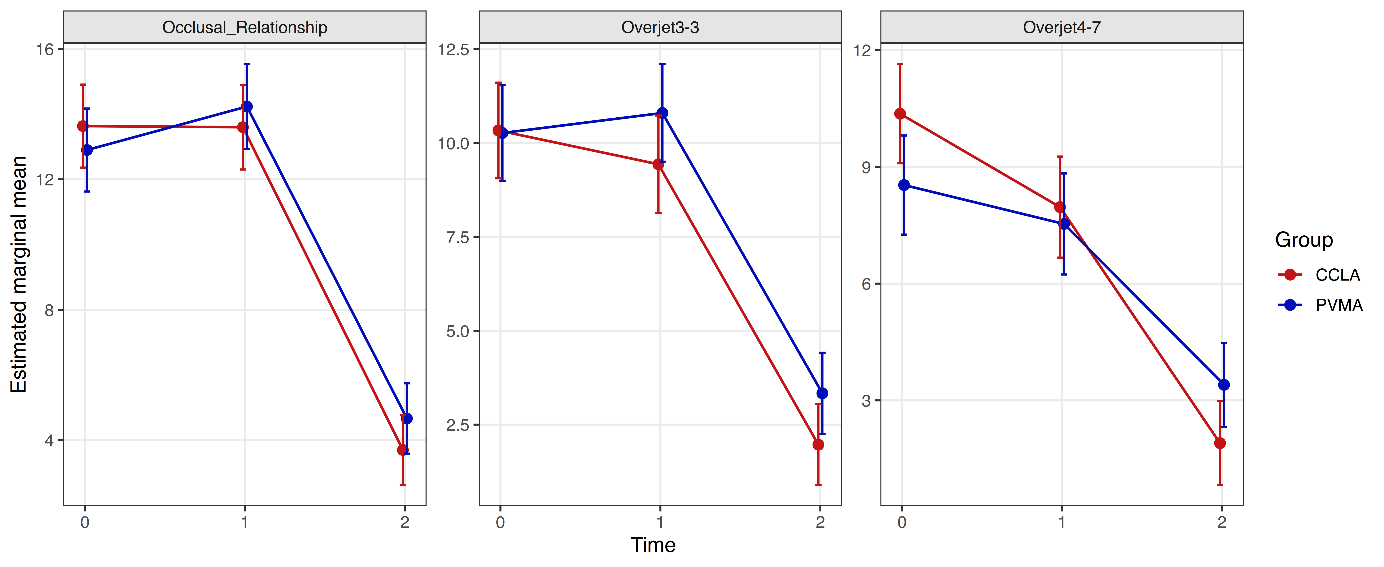


Estimated marginal means with 95% confidence intervals for the intermaxillary ABO criteria of the maxilla and mandible at initial (0), intermediate (1) and final (2) assessment time points for both treatment groups. The model-based mean estimates are represented by the points, and the error bars show the 95% confidence intervals. Lower ABO scores indicate better treatment quality.
